# Clinical Outcomes and Patient Satisfaction After Ultrasound- and Intracavitary ECG-Guided Totally Implantable Venous Access Port Implantation

**DOI:** 10.3390/healthcare14111565

**Published:** 2026-06-03

**Authors:** Sezen Kumaş Solak, Ceren Gür, Serdar Demirgan, Hatice Feyizi, Ali Özalp, Rasim Onur Karaoğlu

**Affiliations:** 1 Department of Anesthesiology, Bağcılar Training and Research Hospital, University of Health Sciences, Istanbul 34203, Turkey; serdardemirgan@hotmail.com (S.D.); haticefeyizi@gmail.com (H.F.); aliozalp1164@gmail.com (A.Ö.); rasimonurkaraoglu@gmail.com (R.O.K.); 2 Department of Internal Medicine, Bağcılar Training and Research Hospital, University of Health Sciences, Istanbul 34203, Turkey; cerencalti@yahoo.com

**Keywords:** totally implantable venous access port (TIVAP), catheter tip positioning, patient satisfaction, catheter-related complications, oncology patients

## Abstract

*Background/Objectives:* Totally implantable venous access ports (TIVAPs) are commonly used in oncology for long-term venous access, but catheter tip malpositioning can lead to complications. Intracavitary electrocardiography (IC-ECG) provides real-time tip guidance, although data on clinical outcomes and patient satisfaction remain limited. This study evaluates postoperative complications, catheter function, and patient comfort and satisfaction after TIVAP implantation using ultrasound and IC-ECG guidance. *Methods:* This retrospective single-center study included adults with solid cancers who received TIVAP implants for chemotherapy from June 2021 to September 2024. Procedures used the right internal jugular vein, with ultrasound guidance and the Seldinger technique; catheter tip placement was guided by IC-ECG. Chest X-rays were obtained to assess for early complications and tip position. Primary outcomes were comfort and satisfaction; secondary outcomes included catheter tip position, catheter complications, and usability. *Results:* Among 213 screened patients, 192 were included (79 females, 113 males; mean age 60.83 ± 12.19 years). The catheter tip was located within the target zone in 149 patients (77.6%). Success rates were 98.44% (*n* = 189/192) for blood aspiration, 97.90% (*n* = 186/190) for port ease of use, 98.95% (*n* = 189/191) for overall satisfaction, and 98.43% (*n* = 188/191) for willingness to recommend. Discomfort occurred in 6.25% (*n* = 12/192) of patients. Comfort improved significantly from day1 to day7 (*p* < 0.001). Complication rates included hematoma (1.56%, *n* = 3), pneumothorax (0.52%, *n* = 1), venous thrombosis (1.56%, *n* = 3), local infection (2.08%, *n* = 4), systemic infection (0.52%, *n* = 1), and catheter occlusion (2.08%, *n* = 4). Catheter blood aspiration success was 93.75% (*n* = 180/192). *Conclusions:* In this retrospective cohort, TIVAP implantation via the right internal jugular vein under ultrasound and IC-ECG guidance was associated with low complication rates, favorable catheter function, and high patient satisfaction. Prospective, multicenter, and comparative studies are needed to determine whether ECG-guided tip positioning improves long-term clinical and patient-centered outcomes.

## 1. Introduction

Totally implantable venous access ports (TIVAPs) are subcutaneously implanted closed venous access systems widely used in contemporary oncology for long-term chemotherapy, parenteral nutrition, and repeated blood sampling, particularly in patients with poor peripheral venous access or those requiring prolonged intravenous treatment [1,2]. Compared with external central venous catheters and peripherally inserted central catheters, TIVAPs offer lower infection rates, greater patient comfort, and superior quality of life during treatment [1,2]. Reported success rates for TIVAP implantation exceed 95% in experienced centers, with overall complication rates ranging from 5% to 15% depending on access route, operator experience, and patient characteristics [2,3]. However, safe implantation and accurate catheter tip positioning remain essential to maximize clinical benefit and minimize device-related harm [1,2].

Despite their well-established advantages, TIVAPs are associated with a spectrum of early and late complications. Early complications include pneumothorax, arterial puncture, local hematoma, and superficial infection, whereas late complications may involve catheter occlusion, venous thrombosis, malposition, arrhythmia, vascular erosion, and, in rare cases, myocardial perforation [2,3]. Among these adverse events, improper catheter tip positioning is particularly important because it is strongly associated with catheter dysfunction, thrombotic complications, vascular wall injury, cardiac irritation, and other potentially serious outcomes [4]. For this reason, accurate determination of catheter length and precise catheter tip positioning are critical components of TIVAP implantation. Although the ideal location of the central venous catheter tip remains somewhat controversial, the lower superior vena cava (SVC) and the cavoatrial junction (CAJ) are generally regarded as the most appropriate and safest target zones for long-term central venous access [5,6]. Radiographic studies have further suggested that the CAJ can be estimated on posteroanterior chest radiography using anatomical landmarks, particularly the carina, and vertebral body unit-based measurements have been proposed to improve standardization of tip assessment [7].

Several techniques are used in clinical practice to estimate catheter length and confirm final tip position, including anatomical landmark-based formulas, chest radiography, fluoroscopic guidance, and ultrasound-assisted approaches [8,9]. Among these methods, intracavitary electrocardiography (IC-ECG) has attracted growing attention because it provides real-time guidance during catheter advancement without radiation exposure. The method relies on characteristic changes in P-wave morphology: as the catheter tip approaches the CAJ, P-wave amplitude progressively increases, and the point of maximal P-wave amplitude is widely accepted as an indicator of optimal tip position [9]. Because of its practicality, real-time feedback, and potential accuracy, IC-ECG has become an attractive alternative to conventional post-procedural confirmation methods in routine venous access practice.

Although IC-ECG guidance has been evaluated for catheter tip positioning in both peripherally inserted central catheters and totally implantable venous access ports, no study to date has simultaneously examined radiographically confirmed tip position, complication rates, and patient-reported comfort and satisfaction following IC-ECG-guided TIVAP implantation via the right internal jugular vein in an oncology population. The present study aims to address this gap.

## 2. Materials and Methods

### 2.1. Study Design, Setting, and Participants

This retrospective, single-center clinical study was conducted at the University of Health Sciences, Bağcılar Training and Research Hospital. Between June 2021 and September 2024, clinical and radiological data from patients who underwent totally implantable venous access port (TIVAP) implantation were analyzed retrospectively. All study endpoints were predefined before protocol submission to the ethics committee.

In this study, patients aged ≥18 years with solid malignancies who underwent TIVAP implantation for chemotherapy were screened for eligibility. Inclusion criteria were the ability to provide informed consent, availability of a postoperative chest X-ray, absence of contraindications to right-sided venous access, complete clinical and radiological data, and completion of the patient satisfaction questionnaire by the patient or, when necessary, a relative. Exclusion criteria comprised pregnancy, pacemaker presence, previous central venous access, thoracic surgical anatomical alterations, mediastinal invasion by lung cancer, significant pulmonary disease, spinal deformity, and prior spinal surgery. Of 213 eligible patients, 192 were included in the final analysis; 21 were excluded because of refusal to participate or lack of written informed consent (*n* = 7), non-response to the satisfaction questionnaire (*n* = 5), or incomplete clinical or radiological data (*n* = 9). All patients were followed for catheter-related complications for a minimum of 6 months. The median follow-up duration was 324 days (interquartile range [IQR]: 252–412 days; range: 182–577 days), and no patients were lost to follow-up. Recorded variables included demographic, clinical, and procedural factors such as age, sex, body mass index (BMI), American Society of Anesthesiologists (ASA) classification, comorbidities, malignancy type, catheterization duration, catheter tip position, arrhythmias, number of attempts, and ultrasound guidance.

### 2.2. Totally Implantable Venous Access Port (TIVAP) Implantation Procedure

All TIVAP procedures were performed by two experienced anesthesiologists with equivalent procedural experience in a fully equipped operating room under sterile conditions. Standard monitoring, including electrocardiography, noninvasive blood pressure, and pulse oximetry, was used throughout the procedure. Patients received sedoanalgesia and local anesthesia with 2% lidocaine infiltration at the puncture and port pocket sites. No general anesthesia or deep sedation was administered, and all patients remained conscious and cooperative throughout the procedure.

**Figure 1:**
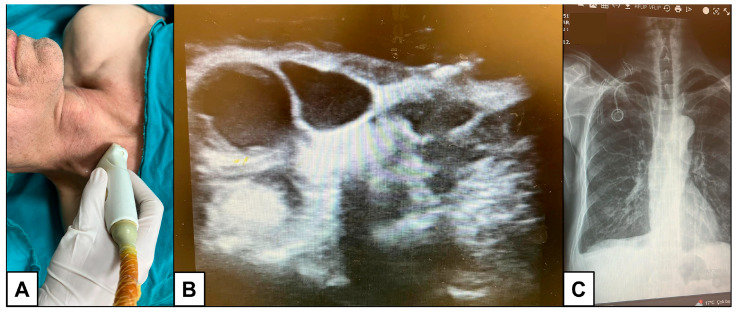
Ultrasound-guided right internal jugular vein access and post-procedural catheter confirmation after TIVAP implantation. (**A**) Clinical view showing patient positioning and ultrasound probe placement over the right cervical region to identify the internal jugular vein before venous puncture. (**B**) Transverse ultrasonographic image of the neck demonstrating the target internal jugular vein and the adjacent carotid artery during real-time vascular assessment prior to cannulation. (**C**) Post-procedural chest radiograph showing the implanted totally implantable venous access port (TIVAP) and catheter, with the tip located in the central venous system at the superior vena cava/cavoatrial junction.

Before implantation, a detailed medical history was obtained, and routine preoperative investigations, including coagulation parameters, complete blood count, electrocardiography, and vascular ultrasonography, were performed. In all cases, the PORT-A-CATH system (Polysite 4000, Vygon, France) was used. Patients were placed in a 10° Trendelenburg position with their head gently rotated to the left. Venous access was obtained exclusively via the right internal jugular vein under real-time ultrasound guidance (Esaote MyLab Five, Genoa, Italy) using the Seldinger technique (Figure 1).

Catheter tip positioning was guided by intracavitary electrocardiography (IC-ECG). During catheter advancement, continuous ECG monitoring was performed by connecting the catheter to the ECG lead system. The catheter tip position was determined by real-time changes in P-wave morphology. As the catheter approached the cavoatrial junction (CAJ), P-wave amplitude progressively increased, and the point of maximal P-wave amplitude was accepted as the optimal catheter tip position. Slight withdrawal was performed when biphasic or negative P-waves were observed, indicating entry into the right atrium.

**Figure 2:**
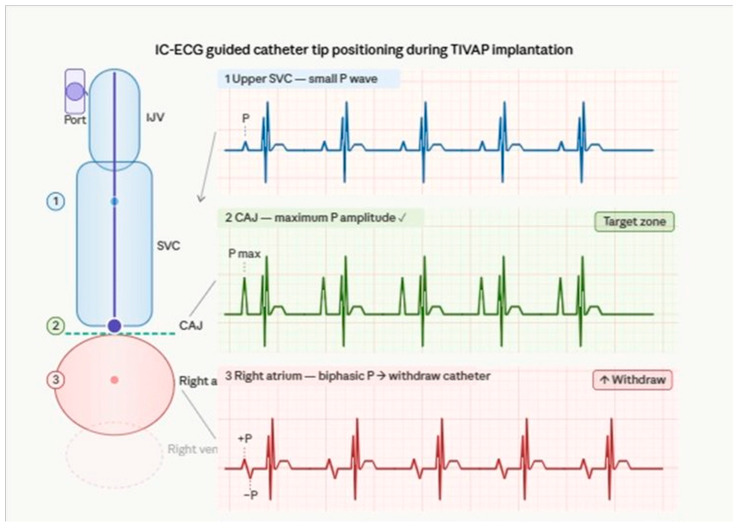
Schematic representation of intracavitary electrocardiography (IC-ECG)-guided catheter tip positioning during totally implantable venous access port (TIVAP) implantation. As the catheter tip advances from the upper superior vena cava (SVC) toward the cavoatrial junction (CAJ), intracavitary P-wave amplitude progressively increases (Strip 1). Maximum P-wave amplitude indicates optimal catheter tip position at the CAJ (Strip 2, target zone). Biphasic or negative P-waves indicate entry into the right atrium, prompting slight withdrawal until maximal P-wave amplitude is restored (Strip 3). CAJ: cavoatrial junction; SVC: superior vena cava; IJV: internal jugular vein.

A straight subcutaneous tunnel was created to prevent catheter kinking, and catheter mobility was confirmed before port attachment. A 3 cm incision parallel to the clavicle was made with a No. 15 scalpel blade, and blunt dissection was performed to create an adequate port pocket. The chamber was placed subcutaneously below the puncture site, and catheter function and stability were confirmed before closure. A postoperative chest radiograph was obtained in all patients to assess early complications and confirm catheter tip position. Radiographic assessment of catheter tip position was performed independently by two experienced clinicians, using the carina as the primary anatomical landmark. The cavoatrial junction was estimated relative to the carina on posteroanterior chest radiography, and the distance between the catheter tip and the cavoatrial junction was measured in millimeters. Interobserver agreement was assessed using Cohen’s kappa coefficient; the two observers agreed in 184 of 192 cases (95.8%), yielding a kappa of 0.880, indicating almost perfect agreement (Figure 1). The IC-ECG technique and corresponding intracavitary P-wave changes during catheter advancement are illustrated in Figure 2.

### 2.3. Measures and Data Collection

Demographic and baseline clinical characteristics included age, sex, height, weight, BMI, ASA physical status, comorbidities, and cancer type. Clinical data, perioperative variables, postoperative complications, and patient satisfaction were obtained from the institutional electronic database and, when necessary, supplemented with telephone-based questionnaires administered to patients or their relatives.

Postoperative catheter-related complications included hematoma, pneumothorax, venous thrombosis, local or systemic infection, catheter occlusion, inability to aspirate blood, and local discomfort. Complications were classified as early (within 30 days of implantation) or late (beyond 30 days). Hematoma was defined as clinically evident blood collection at the port pocket or puncture site; cases requiring surgical intervention were recorded separately from those managed conservatively. Pneumothorax was diagnosed on postoperative chest radiography and classified according to whether observation alone or intervention (aspiration or chest tube drainage) was required. Venous thrombosis was diagnosed by Doppler ultrasonography in patients presenting with ipsilateral limb swelling, pain, or clinical suspicion. Local infection was defined as erythema, warmth, swelling, or purulent discharge at the port pocket or exit site in the absence of systemic signs. Systemic infection was defined as bacteremia or sepsis temporally associated with port use, confirmed by positive blood cultures drawn through the port or peripheral vein. Catheter occlusion was defined as inability to infuse fluids through the port despite positional maneuvers and urokinase instillation. Inability to aspirate blood was defined as failure to withdraw blood from the port during routine access attempts, in the absence of infusion difficulties. Patient comfort was assessed using a six-point ordinal discomfort scale on postoperative day1 (D1) and day7 (D7), graded from 0 to 5: Grade 0, no discomfort; Grade 1, extremely mild discomfort; Grade 2, mild discomfort; Grade 3, moderate discomfort; Grade 4, marked discomfort; and Grade 5, severe discomfort. This scale was adapted from a previously validated instrument used in TIVAP comfort assessment studies [10].

Patient satisfaction was evaluated with a structured questionnaire assessing ease of blood aspiration, cosmetic appearance, discomfort during daily activities and sleep, difficulty with needle insertion, ease of port use, overall satisfaction, and willingness to recommend the device.

### 2.4. Ethical Considerations

The study protocol was reviewed and approved by the Institutional Review Board of Medipol University Hospital (IRB No. 1111/28.11.2024). The study was conducted in accordance with the principles of the Declaration of Helsinki. Written informed consent for the TIVAP implantation procedure was obtained from all patients at the time of the procedure as part of routine clinical care. Additional verbal consent for telephone questionnaire participation was obtained prior to questionnaire administration. The retrospective use of clinical data was approved by the Institutional Review Board.

### 2.5. Statistical Analysis

Statistical analyses were conducted using GraphPad Prism version 10.6 (La Jolla, CA, USA). Continuous variables were reported as mean ± standard deviation or median (interquartile range), as appropriate, and categorical variables were presented as counts and percentages. Normality was assessed with the Shapiro–Wilk test. The Wilcoxon signed-rank test was used to compare paired ordinal comfort scores between postoperative day1 and day7. The Mann–Whitney U test was used for independent two-group comparisons of continuous variables, including comparisons between patients with and without complications. Categorical variables were compared using Fisher’s exact test. A *p*-value of less than 0.05 was considered statistically significant. Spearman’s rank correlation coefficient was used to assess associations between continuous variables, given the non-normal distribution of all continuous measures as confirmed by the Shapiro–Wilk test.

As this was a retrospective observational study, a formal a priori power calculation was not performed. The sample size was determined by the number of eligible patients who underwent TIVAP implantation at the study center during the defined study period. Posthoc power analysis indicated that a sample of 192 patients provided greater than 90% power to detect a clinically meaningful difference of 15 percentage points in patient satisfaction rates (e.g., 95% vs. 80%) at a two-sided significance level of 0.05, which is consistent with the primary outcomes of this study.

## 3. Results

A total of 213 patients were initially assessed for eligibility, and 192 patients were included in the final analysis after application of the inclusion and exclusion criteria. The study population consisted of 79 females (41.15%) and 113 males (58.85%). The mean age was 60.83 ± 12.19 years, with a median age of 62 years (range, 18–88 years). Mean height, weight, and body mass index (BMI) were 166.60 ± 8.39 cm, 70.83 ± 14.57 kg, and 25.49 ± 4.77 kg/m^2^, respectively. With regard to preoperative physical status, ASA II was the most common classification (46.35%), followed by ASA I (33.33%) and ASA III (19.79%), whereas only one patient (0.52%) was classified as ASA IV (Table 1).

Among comorbid conditions, hypertension (HT) was the most frequent, affecting 68 patients (35.42%), followed by diabetes mellitus (DM) in 49 patients (25.52%) and obesity in 32 patients (16.67%). Ischemic heart disease (IHD) was present in 17 patients (8.85%), whereas hypo-/hyperthyroidism was recorded in 9 patients (4.69%). Other comorbidities, including heart failure (HF), asthma, chronic obstructive pulmonary disease (COPD), cerebrovascular accident (CVA), and chronic kidney disease (CKD), were observed less frequently (Table 2).

Regarding malignancy type, colorectal cancer was the most common diagnosis, observed in 78 patients (40.63%), followed by stomach cancer in 60 patients (31.25%). Breast cancer was present in 21 patients (10.94%), and pancreatic cancer in 15 patients (7.81%). Less frequent malignancies included esophagus, larynx, and lung cancers (each 2.08%), as well as liver and ovarian cancers (each 1.04%) (Table 3).

Patient-reported outcomes indicated high acceptance and usability of the implanted port system. Most patients reported that blood aspiration from the port was easy (98.44%) and that the port was easy to use (97.90%). Cosmetic dissatisfaction was uncommon, with only 9.90% of patients reporting the port was visually disturbing. Similarly, discomfort during daily activities and during sleep was reported by only 6.25% of patients in each category. Difficulty with needle insertion for chemotherapy was noted in 5.79% of patients. Overall satisfaction with the port was very high (98.95%), and 98.43% of patients stated they would recommend the device to other patients (Table 4).

Catheter tip position was assessed on postoperative chest radiography in all 192 patients. Among these, the catheter tip was located within the target zone in 149 patients (77.6%), of whom 107 (55.7%) were positioned at the CAJ or lower SVC without right atrial entry and 42 (21.9%) were at the CAJ boundary with right atrial entry noted. Among the 43 patients (22.4%) outside the target zone, 8 (4.2%) had tips in the upper or mid SVC and 35 (18.2%) had tips in the deep right atrium. The median distance between the catheter tip and the cavoatrial junction was 0 mm (IQR: −9 to +9 mm) (Table 5).

When complications were compared according to catheter tip position, no statistically significant difference was observed between patients with tips in the target zone and those outside the target zone for hematoma (2.8% vs. 2.5%, *p* = 1.000) or pneumothorax (0.7% vs. 0.0%, *p* = 1.000). These comparisons were performed using Fisher’s exact test.

Patient comfort scores were also evaluated on postoperative day1 and day7. The median comfort score was 0 on both days; however, the range decreased from 0–4 on day1 to 0–3 on day7, and this difference was statistically significant (*p* < 0.001). These findings indicate that patient comfort remained favorable in the early postoperative period and improved further by the seventh postoperative day (Table 6).

Postoperative catheter-related complications were infrequent overall. Hematoma developed in 3 patients (1.56%), pneumothorax in 1 patient (0.52%), and venous thrombosis in 3 patients (1.56%). Local infection and catheter occlusion were each observed in 4 patients (2.08%), whereas systemic infection occurred in 1 patient (0.52%). Blood aspiration from the catheter was successful in 180 patients (93.75%), while inability to aspirate blood was documented in 12 patients (6.25%). Mortality was recorded in 45 patients (23.44%) during follow-up, whereas 147 patients (76.56%) were alive at the time of assessment (Table 7). The median follow-up duration was 324 days (IQR: 252–412 days; range: 182–577 days).

To identify potential predictors of complications, patients with and without postoperative complications were compared with respect to demographic and clinical variables. No statistically significant differences were observed between groups in terms of age (median 60 vs. 61 years, *p* = 0.220), BMI (25.4 vs. 24.8 kg/m^2^, *p* = 0.704), or duration of surgery (30 vs. 26 min, *p* = 0.078). Similarly, the rates of ASA III–IV classification (14.3% vs. 19.8%, *p* = 1.000), diabetes mellitus (14.3% vs. 25.2%, *p* = 0.685), obesity (0.0% vs. 16.8%, *p* = 0.603), multiple venipuncture attempts (42.9% vs. 27.5%, *p* = 0.402), and metastatic disease (71.4% vs. 43.5%, *p* = 0.247) did not differ significantly between patients with and without complications. Continuous variables were compared using the Mann–Whitney U test and categorical variables using Fisher’s exact test.

Normality testing with the Shapiro–Wilk test revealed non-normal distributions for all continuous variables, including age (W = 0.980, *p* = 0.001), BMI (W = 0.971, *p* < 0.001), IJV diameter (W = 0.972, *p* < 0.001), and procedure duration (W = 0.922, *p* < 0.001). Spearman correlation analysis demonstrated a significant positive association between the number of venipuncture attempts and procedure duration (r = 0.231, *p* < 0.001), indicating that procedures requiring multiple puncture attempts were associated with longer operative times. A weak but statistically significant positive correlation was observed between age and IJV diameter (r = 0.125, *p* = 0.044) and between BMI and IJV diameter (r = 0.130, *p* = 0.037).

## 4. Discussion

This study evaluated IC-ECG-guided TIVAP implantation via the right internal jugular vein in a real-world oncology cohort, simultaneously addressing catheter tip position, complication rates, and patient-reported outcomes—dimensions that have rarely been examined together in the existing literature. The observed target zone placement rate of 77.6%, low complication rates, and satisfaction exceeding 98% add to the evidence base supporting standardized ultrasound- and IC-ECG-guided TIVAP implantation as a practical approach in oncology practice. As this was a retrospective single-center study without a comparator group, these findings reflect an association with favorable outcomes rather than evidence of causality.

Accurate catheter tip positioning remains a central determinant of TIVAP safety and long-term performance. Contemporary reviews and expert consensus continue to identify the lower third of the superior vena cava (SVC) and the cavoatrial junction (CAJ) as the preferred target zones for central venous access devices, because these locations optimize hemodilution and may reduce endothelial irritation, thrombosis, dysfunction, and cardiac complications [11,12]. This issue is clinically relevant because tip misplacement remains common in central venous catheter practice and is associated with avoidable adverse events [13]. In this study, IC-ECG was used to identify the CAJ in real time through maximal P-wave amplitude, while postoperative chest radiography was retained as a confirmatory tool. This combined approach aligns with current practice, and randomized evidence suggests that IC-ECG outperforms surface landmark measurement for tip localization [14]. Recent comparative studies have further demonstrated that IC-ECG-guided tip positioning achieves equivalent or superior accuracy compared with fluoroscopic guidance in TIVAP implantation, with favorable safety profiles in large oncology cohorts [15,16]. Similarly, previous studies have suggested that ultrasound with IC-ECG guidance may reduce reliance on routine postprocedural chest radiography for tip position confirmation alone, although radiography still has value in detecting immediate complications such as pneumothorax [17,18].

The low complication rates observed in this cohort align with the modern TIVAP literature. Reviews and recent oncology-focused studies indicate that complication rates are minimized when ports are inserted by experienced operators using ultrasound-guided venous access, standardized sterile technique, and structured tip position confirmation methods [14,19]. In this series, pneumothorax occurred in only 0.52% of patients and hematoma in 1.56%, suggesting that right internal jugular access under real-time ultrasound guidance was associated with low rates of mechanical access-related harm. This interpretation is anatomically plausible because the right internal jugular route provides a relatively direct path to the SVC and avoids pinch-off, which is more commonly associated with subclavian access [14,20]. Nevertheless, because this study did not directly compare venous access sites, this explanation should be interpreted cautiously.

Thrombotic, infectious, and functional complications were also infrequent. Venous thrombosis was observed in 1.56% of patients, local infection in 2.08%, systemic infection in 0.52%, and catheter occlusion in 2.08%. These findings are clinically important because thrombosis, infection, and dysfunction remain leading causes of unplanned interventions, device failure, or premature port removal in oncology populations [14,19]. Functional performance was likewise favorable: catheter aspiration was successful in 93.75% of patients, and nearly all patients subjectively reported that blood could be drawn easily from the port. This concordance between objective catheter function and patient perception strengthens the practical relevance of our findings. At the same time, the small subset of patients with absent aspiration or occlusion remains clinically meaningful, since these events may reflect fibrin sheath formation, partial tip migration, or other maintenance-related problems that cannot be fully clarified by the present dataset [14,19]. Because serial imaging was not performed, we were unable to determine whether late changes in tip position contributed to these events.

An important strength of the present study is the inclusion of patient-reported outcomes alongside clinical endpoints. Most patients described the port as easy to use, cosmetically acceptable, and minimally disruptive to daily activities or sleep. Overall satisfaction was 98.95%, and 98.43% of patients stated they would recommend the device to others. These findings are highly consistent with previous studies showing that implantable ports are generally associated with favorable quality of life, greater treatment convenience, and strong patient acceptance compared with more externally visible or maintenance-intensive vascular access devices [21,22,23]. The significant improvement in comfort scores from postoperative day 1 to day 7 further suggests that early post-procedural discomfort decreases rapidly, supporting the intervention’s short-term tolerability. In practical terms, this is important because the success of TIVAP use in oncology depends not only on technical safety but also on whether patients perceive the device as acceptable in daily life. Several methodological factors may have influenced the observed satisfaction rates and should be considered when interpreting these findings. First, the telephone-based interview format may have introduced social desirability bias, as patients may be reluctant to express dissatisfaction directly to their treating team. Second, relatives completed the questionnaire on behalf of some patients, which may not accurately reflect the patient’s own experience. Third, oncology patients who are dependent on their port for chemotherapy delivery may perceive the device more favorably due to its perceived necessity, regardless of comfort or cosmetic concerns. Fourth, the absence of a validated, standardized patient-reported outcome instrument limits direct comparison with other studies. Taken together, these factors suggest that the very high satisfaction rates reported here should be interpreted with appropriate caution.

The mortality rate in our cohort was 23.44% during follow-up. This finding should be interpreted in the context of the underlying malignancy burden rather than as a port-specific safety signal. The current tables do not distinguish cancer-related deaths from catheter-related deaths, and the available results do not support attributing mortality directly to the TIVAP procedure. Cause of death was not available for all patients; therefore, cancer-related versus device-related mortality could not be formally distinguished. For this reason, mortality is best reported as an overall cohort outcome rather than as a direct device complication.

Several limitations should be acknowledged. First, the retrospective, single-center design limits causal inference and generalizability. Second, no direct comparison was made with fluoroscopy-guided placement, landmark-based estimation, or alternative tip-confirmation techniques. Third, although the study objective included catheter tip positioning, the results presented here do not provide a stratified comparison of optimally positioned versus malpositioned catheters with respect to complications or satisfaction; therefore, strong claims about the effect of tip position on outcomes in this cohort would be premature. Fourth, longitudinal catheter tip migration was not systematically assessed. In addition, cause-specific mortality data were not systematically available; therefore, overall mortality outcomes could not be directly attributed to TIVAP-related complications. Additionally, operator-level complication data were not analyzed separately, as all procedures were performed by two anesthesiologists with equivalent experience; individual operator volume and learning curve effects therefore could not be assessed. Finally, the comfort scale used in this study has not undergone formal psychometric validation, which limits direct comparability with other patient-reported outcome instruments.

Despite limitations, the study has notable strengths. It reflects real-world practice in a standardized procedural setting, with all devices placed via the right internal jugular vein under ultrasound and IC-ECG guidance. It also combines objective postoperative outcomes with patient-reported comfort and satisfaction, offering a broader, more patient-centered evaluation of TIVAP performance than complication-only studies. Future prospective multicenter studies should directly compare IC-ECG with fluoroscopy and other confirmation strategies, ideally using predefined radiographic or echocardiographic standards for the CAJ. Stratified analyses by final tip position, access route, and cancer type would help identify which patients benefit most from ECG-guided implantation. Longer follow-up with serial imaging may also clarify whether late tip migration contributes to dysfunction, thrombosis, or aspiration failure.

## 5. Conclusions

TIVAP implantation via the right internal jugular vein under ultrasound and IC-ECG guidance was associated with low rates of catheter-related complications, high functional usability, and very high patient satisfaction in this retrospective cohort. Patient comfort improved significantly during the first postoperative week. These findings suggest that IC-ECG-guided TIVAP placement may represent a practical and well-tolerated strategy for oncology patients. However, because this was a retrospective, single-center study without a comparator group or stratified analysis by tip position, the clinical implications should be interpreted cautiously. Prospective, multicenter, and comparative studies are needed to determine whether ECG-guided optimization of catheter tip position translates into measurable reductions in complications and improved long-term patient-centered outcomes.

## Figures and Tables

**Table 1:** Demographic data and clinical features.

**Age**	***Min–Max (Median)***	18–88 (62)
	***Mean ± SD***	60.83 ± 12.19
**Gender**	***Female/n (%)***	79 (41.15)
	***Male/n (%)***	113 (58.85)
**Height (cm)**	***Min–Max (Median)***	140–190 (167.5)
	***Mean ± SD***	166.60 ± 8.39
**Weight (kg)**	***Min–Max (Median)***	38–130 (70)
	***Mean ± SD***	70.83 ± 14.57
**BMI**	***Min–Max (Median)***	16.41–44.98 (24.789)
	***Mean ± SD***	25.49 ± 4.77
**ASA**	***I/n (%)***	64 (33.33)
	***II/n (%)***	89 (46.35)
	***III/n (%)***	38 (19.79)
	***IV/n (%)***	1 (0.52%)

BMI: body mass index; ASA: American Society of Anesthesiologists; SD: standard deviation.

**Table 2:** Comorbidities.

Comorbidity *	*n* (%)	Comorbidity *	*n* (%)
- Asthma	6 (3.13)	- HT	68 (35.42)
- CKD	1 (0.52)	- Hypo-/Hyperthyroidism	9 (4.69)
- CVA	3 (1.56)	- IHD	17 (8.85)
- DM	49 (25.52)	- COPD	5 (2.60)
- HF	7 (3.65)	- Obesity	32 (16.67)

* The prevalence rates of comorbidities (%) were determined in relation to the totalstudy population (*n*). HT: hypertension; DM: diabetes mellitus; IHD: ischemic heart disease; HF: heart failure; CKD: chronic kidney disease; CVA: cerebrovascular accident; COPD: chronic obstructive pulmonary disease.

**Table 3:** Cancer type distribution.

Cancer Type	*n* (%)	Cancer Type	*n* (%)
- Breast	21 (10.94)	- Lung	4 (2.08)
- Colorectal	78 (40.63)	- Stomach	60 (31.25)
- Esophagus	4 (2.08)	- Pancreas	15 (7.81)
- Larynx	4 (2.08)	- Other	2 (1.04)
- Liver	2 (1.04)	- Ovary	2 (1.04)

**Table 4:** Patient Questionnaire on Port Comfort and Usability.

**Is it easy to draw blood from the port? (*n* = 192)**	***Yes/n (%)***	189 (98.44)
	***No/n (%)***	3 (1.56)
**Is the port cosmetically/visually disturbing? (*n* = 192)**	***Yes/n (%)***	19 (9.90)
	***No/n (%)***	173 (90.10)
**Is it uncomfortable during daily activities? (*n* = 192)**	***Yes/n (%)***	12 (6.25)
	***No/n (%)***	180 (93.75)
**Is it uncomfortable while sleeping? (*n* = 192)**	***Yes/n (%)***	12 (6.25)
	***No/n (%)***	180 (93.75)
**Have you experienced any difficulty inserting the needle for chemotherapy? (*n* = 190)**	***Yes/n (%)***	11 (5.79)
	***No/n (%)***	179 (94.21)
**Is the port easy to use? (*n* = 190)**	***Yes/n (%)***	186 (97.90)
	***No/n (%)***	4 (2.10)
**Are you satisfied with the port overall? (*n* = 191)**	***Yes/n (%)***	189 (98.95)
	***No/n (%)***	2 (1.05)
**Would you recommend it to other patients? (*n* = 191)**	***Yes/n (%)***	188 (98.43)
	***No/n (%)***	3 (1.57)

Response rates varied by item due to missing data; denominators are reported for each question.

**Table 5:** Detailed Catheter Tip Position on Postoperative Chest Radiography.

Catheter Tip Position	*n* (%)
**Target zone**	
CAJ/lower SVC (no right atrial entry)	107 (55.7)
CAJ boundary (right atrial entry noted)	42 (21.9)
** Subtotal**	**149 (77.6)**
**Outside target zone**	
Upper/mid-SVC	8 (4.2)
Deep right atrium	35 (18.2)
** Subtotal**	**43 (22.4)**
**Total**	**192 (100)**

CAJ: cavoatrial junction; SVC: superior vena cava; Target zone defined as lower SVC or cavoatrial junction. One patient with catheter tip extending into the right ventricle was repositioned intraoperatively and is included in the deep right atrium category.

**Table 6:** Patient Comfort Scale.

Variable	Min–Max (Median)
Patient comfort scale D1	0–4 (0)
Patient comfort scale D7	0–3 (0)
***p* value**	<0.001 ****

D1: postoperative day 1; D7: postoperative day 7, ****: *p* < 0.001.

**Table 7:** Postoperative Data: Catheter-Related Complications.

**Hematoma**	***Yes/n (%)***	3 (1.56)
	***No/n (%)***	189 (98.44)
**Pneumothorax**	***Yes/n (%)***	1 (0.52)
	***No/n (%)***	191 (99.48)
**Venous thrombosis**	***Yes/n (%)***	3 (1.56)
	***No/n (%)***	189 (98.44)
**Infection (local)**	***Yes/n (%)***	4 (2.08)
	***No/n (%)***	188 (97.92)
**Infection (systemic)**	***Yes/n (%)***	1 (0.52)
	***No/n (%)***	191 (99.48)
**Catheter occlusion**	***Yes/n (%)***	4 (2.08)
	***No/n (%)***	188 (97.92)
**Blood aspiration from catheter**	***Yes/n (%)***	180 (93.75)
	***No/n (%)***	12 (6.25)

All values represent number of patients (%).

## Data Availability

The data that support the findings of this study are available from the corresponding author upon reasonable request.

## Abbreviations

The following abbreviations are used in this manuscript:

ASA	American Society of Anesthesiologists
BMI	Body mass index
CAJ	Cavoatrial junction
CKD	Chronic kidney disease
COPD	Chronic obstructive pulmonary disease
CVA	Cerebrovascular accident
CXR	Chest X-ray
DM	Diabetes mellitus
HF	Heart failure
HT	Hypertension
IC-ECG	Intracavitary electrocardiography
IHD	Ischemic heart disease
IJV	Internal jugular vein
PACS	Picture archiving and communication system
RA	Right atrium
SVC	Superior vena cava
TIVAP	Totally implantable venous access port
